# Characterization and Antibiotic Resistance of *Listeria monocytogenes* Strains Isolated from Greek Myzithra Soft Whey Cheese and Related Food Processing Surfaces over Two-and-a-Half Years of Safety Monitoring in a Cheese Processing Facility

**DOI:** 10.3390/foods12061200

**Published:** 2023-03-12

**Authors:** Nikolaos D. Andritsos, Marios Mataragas

**Affiliations:** 1 Department of Food Science and Technology, School of Agricultural Sciences, University of Patras, 2 G. Seferi Str., GR-301 00 Agrinio, Greece; 2 Department of Dairy Research, Institute of Technology of Agricultural Products, Hellenic Agricultural Organization “DIMITRA”, 3 Ethnikis Antistaseos Str., GR-452 21 Ioannina, Greece

**Keywords:** antimicrobial resistance, cheese processing environment, *Listeria monocytogenes*, Myzithra, virulence genes, whey cheeses, whole-genome sequencing

## Abstract

Listeriosis is a serious infectious disease with one of the highest case fatality rates (*ca.* 20%) among the diseases manifested from bacterial foodborne pathogens in humans, while dairy products are often implicated as sources of human infection with *Listeria monocytogenes*. In this study, we characterized phenotypically and genetically by whole-genome sequencing (WGS) 54 *L. monocytogenes* strains isolated from Myzithra, a traditional Greek soft whey cheese (48 isolates), and swabs collected from surfaces of a cheese processing plant (six isolates) in the Epirus region of Greece. All but one strain of *L. monocytogenes* belonged to the polymerase chain reaction (PCR) serogroups IIa (16.7%) and IIb (81.5%), corresponding to serotypes 1/2a, 3a and 1/2b, 3b, 7, respectively. The latter was identified as a PCR-serogroup IVb strain (1.8%) of serotypes 4b, 4d, 4e. Bioinformatics analysis revealed the presence of five sequence types (STs) and clonal complexes (CCs); ST1, ST3, ST121, ST 155, ST398 and CC1, CC3, CC121, CC155, CC398 were thus detected in 1.9, 83.3, 11.0, 1.9, and 1.9% of the *L. monocytogenes* isolates, respectively. Antibiograms of the pathogen against a panel of seven selected antibiotics (erythromycin, tetracycline, benzylpenicillin, trimethoprim-sulfamethoxazole, ampicillin, ciprofloxacin, and meropenem) showed that 50 strains (92.6%), the six surface isolates also included, were intermediately resistant to ciprofloxacin and susceptible to the rest of the six antimicrobial agents tested, whereas strong resistance against the use of a single from three implicated antibiotics was recorded to four strains (7.4%) of the pathogen isolated from Myzithra cheese samples. Thence, the minimum inhibitory concentrations (MICs) were determined for erythromycin (MIC = 0.19 μg/mL), ciprofloxacin (MIC ≥ 0.19 μg/mL), and meropenem (MIC = 0.64 μg/mL), and finally, just one strain was deemed resistant to the latter antibiotic. The phylogenetic positions of the *L. monocytogenes* strains and their genetic variability were determined through WGS, whilst also stress response and virulence gene analysis for the isolates was conducted. Findings of this work should be useful as they could be utilized for epidemiological investigations of *L. monocytogenes* in the food processing environment, revealing possible contamination scenarios, and acquired antimicrobial resistance along the food production chain.

## 1. Introduction

*Listeria monocytogenes* is the etiological agent of listeriosis disease in both humans and animals. The ubiquitous character of the bacterium makes it widespread in both the natural environment and the food processing environment, thus easily contaminating fresh produce (e.g., fruits and vegetables) [1,2,3,4,5] and foods of animal origin (e.g., meat and dairy products) [6,7,8,9]. Listeriosis is a serious infectious disease with one of the highest case fatality rates in humans (*ca.* 20%) among the diseases manifesting from bacterial pathogens encountered in foods [10,11,12]. Contrary to the non-invasive form of febrile gastroenteritis (intestinal listeriosis), symptoms of the severe invasive form of the disease occur when the pathogen has spread beyond the intestines of the infected person. They include fever, muscle pain, septicemia, meningitis, and meningoencephalitis, and they usually start within two weeks of eating food contaminated with *L. monocytogenes* [13,14]. Invasive listeriosis affects the most vulnerable segment of the population; YOPI individuals (i.e., young, old, pregnant, and immunocompromised), such as pregnant women and their newborns, infants, elderly people (above 65 years old) and people with a weakened immune system (e.g., patients undergoing treatment for hemodialysis, cancer, or AIDS). In contrast, febrile gastroenteritis is a mild form of listeriosis that normally ends up as a self-limiting infection, affecting mainly otherwise healthy people after ingesting a rather large number of *L. monocytogenes* bacteria from contaminated food products [13,14]. In this case, symptoms usually start within a few days (mostly within 24 h) after the consumption of contaminated food with the pathogen and include diarrhea, fever, headache, and muscle pain [14].

Control of *L. monocytogenes* in the food processing environment is quite difficult due to the pathogen’s ability to tolerate extreme environmental conditions (e.g., acid resistance, heat resistance, and high salt concentrations) [15,16,17,18,19,20,21]; adapt to a wide range of physical/physicochemical stresses (e.g., pH, water activity, and temperature fluctuations) [16,17,19,20]; cope with sublethal stresses induced from treatments with antimicrobials and disinfectants (e.g., plant essential oils or quaternary ammonium compounds (QACs)) [20,21,22,23]; and form biofilms as means of self-preservation [20,24,25]. These factors eventually lead to the persistence of *L. monocytogenes* in food equipment and premises [20,26,27,28].

Animal-originated foods, such as meat, milk, and dairy products, are excellent substrates for microbial proliferation. Most dairy products (e.g., cheese and yogurt) are the result of microbial activity (fermentation) of the native microbiota contained in milk, albeit in the food and cheese production process in particular, microbial pathogens may gain access to the final product and represent a threat to the consumer [29], given their growth potential and survivability in cheese and its products thereof [30,31,32,33,34,35]. *L. monocytogenes* cannot grow in the traditional Greek soft acid-curd cheeses, such as Galotyri and Katiki [35,36], or hard ripened cheeses like traditional Greek Graviera cheese [32,37], although effective control of post-processing listerial cross-contamination is needed in Greek Myzithra, Anthotyros, and Manouri soft whey cheeses [38,39,40]. Thankfully, to the best of the authors’ knowledge, the last documented survey conducted in the Greek retail market, although published a decade ago, indicated a low prevalence of *Listeria* spp. and the absence of positive samples for *L. monocytogenes* in soft cheeses [41]. In strict alignment with those findings, a recent systematic review and meta-analysis conducted with data from the European Food Safety Authority (EFSA) and the scientific literature emphasized the lowest mean prevalence (*ca.* 0.8%) of *L. monocytogenes* in fresh cheeses, like Myzithra, among all other European cheeses (i.e., ripened, veined, smear, and brined) [42].

The vast majority of human listeriosis cases are caused by *L. monocytogenes* serotypes 1/2a (lineage II), 1/2b and 4b (lineage I) [43,44,45]. Nevertheless, more than 50% of human listeriosis outbreaks are associated with lineage I and to be precise most of these outbreaks are attributed to serotype 4b (polymerase chain reaction serogroup IVb; PCR-serogroup IVb), whereas serotype 1/2a (PCR-serogroup IIa) and 1/2b (PCR-serogroup IIb) strains of the pathogen are mostly isolated from foods [45,46] (pp. 50–51). This could be attributed not only to the inherent genetic characteristics [45] and the competitive growth advantage between the strains (PCR-serogroups IIa, IIb and IIc vs. PCR-serogroup IVb) [47] but also to the selective enrichment protocol used for the *L. monocytogenes* detection [48], justifying in this way the overrepresentation of certain serotypes of the pathogen in foods.

Epidemiological studies aim to link the pathogen responsible for the advent of an outbreak, isolated from human clinical samples, with the implicated food commodity in case of a foodborne disease investigation. Many times, food and environmental sampling are established towards the investigation of the above linkage, leading to the deciphering of relevant contamination routes for the pathogen under study [49]. Serotyping and serogrouping of strains are of low discriminatory capacity and offer very little to the epidemiological surveillance of the pathogens, such as *L. monocytogenes* [43]. In this context, laboratory methods with increased discriminatory power, such as pulsed-field gel electrophoresis (PFGE), multilocus sequence typing (MLST) and repetitive element palindromic PCR (rep-PCR) in combination with random amplified polymorphic DNA PCR (RAPD-PCR) analysis, have been utilized for the subtyping of *L. monocytogenes* isolated from foods and the food processing environment [50,51,52]. However, in recent years, whole-genome sequencing (WGS) is gaining more and more ground and is tending to replace PFGE, MLST and rep-PCR with RAPD-PCR fingerprinting of pathogen isolates [53,54], highlighting the population diversity and persistence of *L. monocytogenes* in the environment of the food production facility [28,55,56,57]. Therefore, the objective of this work was to carry out the phenotypic and genomic characterization, as well as the antibiotic resistance profile of *L. monocytogenes* strains isolated from both the cheese processing environment and samples of an end-product (Myzithra cheese). The genetic variability of the strains, along with their phylogenetic positions, were determined by means of WGS. In parallel, a stress response and virulence gene analysis for the isolates was conducted, allowing for a better understanding of *L. monocytogenes’* persistence in the cheese production environment and thus contributing towards improved strategies for the elimination of the pathogen.

## 2. Materials and Methods

### 2.1. Sampling of Cheese and Related Food Processing Surfaces

Environmental and end-product sampling was performed during the production process in a cheese processing facility located in the Epirus region of northwestern Greece. Related cheese processing surfaces and Myzithra, a traditional Greek soft whey cheese manufactured from sheep and/or goat milk, were sampled to detect *L. monocytogenes*. Screening for the pathogen’s presence took place over a period of more than two-and-a-half years of microbiological monitoring of *L. monocytogenes* in the facility (November 2016–July 2019).

Samples of fresh and dried Myzithra end-product comprised of *ca.* 150 g of cheese. Surface sampling was performed according to the protocol of the International Organization for Standardization (ISO 18593) [58,59] by using pre-moistened in half concentration-Fraser broth (½-FB; Biokar Diagnostics, Pantin, France), sterile cotton applicators (AnQing Jiaxin Medical Technology Co., Ltd., Anqing, China) and swabbing 100 cm^2^ (10 cm × 10 cm), with a swabbing movement executed in both horizontal and vertical directions onto the surface and by rotating the applicator between fingers, then placing the swab under aseptic conditions in 10 mL of ½-FB contained in Sterilin ™ plastic round-based tubes with screw cap (Sarstedt, Nümbrecht, Germany). Cheese and surface samples were transported into isothermal boxes with ice packs and were analyzed upon arrival at the laboratory.

### 2.2. L. monocytogenes Isolation

The ISO 11290-1 protocol was used for the detection of *L. monocytogenes* [60,61] from cheese and surface samples with some slight modifications. Briefly, 25 g of Myzithra end-product were transferred aseptically in a stomacher bag and homogenized with 225 mL of ½-FB in a Stomacher blender (BagMixer^®^ 400 W, Interscience, Saint Nom, France). The homogenate was incubated at 30 °C for 24 h. In the case of surface samples, Sterilin™ tubes containing the swabs in 10 mL of ½-FB were placed for incubation at the aforementioned conditions immediately upon arrival at the laboratory. Following the primary enrichment of samples, 0.1 mL of the initial suspension was transferred aseptically to a tube containing 10 mL of full concentration-FB (FB; Biokar Diagnostics) as a secondary enrichment medium. The inoculated FB was incubated then at 37 °C for 24–48 h. Primary and secondary enrichment broths (½-FB and FB) were streaked on duplicate plates of COMPASS^®^ *Listeria* agar (Biokar Diagnostics), its formulation of which is in accordance with the agar *Listeria* according to Ottaviani and Agosti (ALOA) referred in the ISO detection method (ISO 11290-1) [60,61].

ALOA plates were examined visually after incubation at 37 °C for 48 h for the presence of the pathogen indicated by well-isolated blue-green colonies surrounded by an opaque halo. Presumptive *L. monocytogenes* was confirmed through biochemical testing of the isolates after subculturing the selected colonies from ALOA on tryptone soya agar containing 0.6% yeast extract (TSAYE) and incubating at 37 °C for 18 to 24 h. Biochemical tests were carried out on colonies from pure cultures grown on TSAYE plates and comprised of oxidase test, catalase reaction, motility test at 25 °C, hemolysis on sheep blood agar, CAMP test, L-rhamnose and D-xylose utilization. Confirmed *L. monocytogenes* isolates were maintained at −80 °C in brain heart infusion (BHI; LabM, Lancashire, UK) broth with 20% glycerol (Biolife, Milan, Italy). The strains were deposited in the microbial culture collection of Eurofins Athens Analysis Laboratories S.A. (AAL; Microbiology Laboratory, Metamorfosi, Attica, Greece) and received an accession number. Before further use of the isolates, the frozen stock of each strain of the pathogen (Supplementary Table S1) was subcultured twice in BHI broth incubated each time at 37 °C for 24 h and then streaked on TSAYE which was incubated at 37 °C for 18 to 24 h.

### 2.3. Antibiotic Resistance Profiles of L. monocytogenes Strains

Antimicrobial susceptibility and resistance to antibiotics of *L. monocytogenes* cheese and surface isolates were determined using the standardized disk diffusion method as recommended by the European Committee on Antimicrobial Susceptibility Testing (EUCAST) [62]. In brief, 3–4 colonies picked after activation of the pathogen on the TSAYE plate were suspended in 3 mL of 0.85% peptone salt solution or maximum recovery diluent (MRD; Merck, Darmstadt, Germany), and turbidity was adjusted to a 0.5 McFarland standard. The suspension was used to inoculate ready-to-use 9 cm Mueller-Hinton agar plates supplemented with 5% defibrinated horse blood and 20 mg/l β-NAD (MH-F; Bioprepare, Keratea, Attica, Greece). Inoculation of MH–F blood agar with the pathogen was achieved by means of microbial lawn formed for each *L. monocytogenes* strain, using sterile cotton applicators (AnQing Jiaxin Medical Technology Co., Ltd.) moistened in the MRD suspension and applied on the surface of MH–F agar with swabbing movement in both horizontal and vertical directions and by rotating the applicator between fingers, covering in that manner the whole agar surface. Antibiotic disks of seven antimicrobial agents were transferred on the MH–F agar surface with the help of forceps. All disks were supplied by Oxoid (Basingstoke, UK): Erythromycin (E; 15 μg), tetracycline (TE; 30 μg), benzylpenicillin (P; 1 IU), trimethoprim-sulfamethoxazole 1:19 (SXT; 25 μg), ampicillin (AMP; 2 μg), ciprofloxacin (CIP; 5 μg) and meropenem (MEM; 10 μg). After 18 h of incubation at 35 °C, the diameters of inhibition zones around the disks were measured to the nearest integral number (in mm) by using a Vernier caliper with the least count of 0.1 mm. Results for inhibition zone diameters per antibiotic and strain of the pathogen were interpreted as susceptible, intermediate, or resistant *L. monocytogenes* isolates to the respective antimicrobial agent based on the criteria provided by EUCAST [62]. Missing zone diameter breakpoints for *L. monocytogenes* resistance against E and CIP were obtained from those recommended by EUCAST for Gram-positive *Staphylococcus aureus* [62].

Profiles of antibiotic resistance for *L. monocytogenes* strains isolated from cheese and related cheese processing surfaces were complemented with the determination of minimum inhibitory concentrations (MICs) by using the E-test method for those antimicrobial agents inducing resistance of the pathogen, screened through the antimicrobial susceptibility testing (AST) as described above. Following inoculation of MH–F agar as previously described, plastic strips with a gradient concentration of the antibiotic of interest to which the strain showed resistance during AST were placed onto the surface of the agar medium, allowing for the diffusion of the antimicrobial agent into the agar and thus providing, after overnight incubation at 37 °C, an inhibition ellipse where the MIC corresponds to the value at the point of intersection of the growth and inhibition zone with the extremity of the strip. The E-test MICs (in μg/mL or ppm) can be read directly from the upper of the Petri dish [63] (pp. 323–324).

### 2.4. Whole-Genome Sequencing of Bacterial Isolates

#### 2.4.1. DNA Extraction and Sequencing

Total DNA was extracted from the bacterial cells of *L. monocytogenes* strains, cultured in BHI broth and incubated overnight at 37 °C after a purity check on TSAYE plates. The genomic DNA was extracted and sequenced by Novogene Genomics Service (Novogene Co., Ltd., Cambridge, UK), and all procedures involving library preparation, genome assembly, and quality control were performed as described by Syrokou et al. [64]. To this end, the complete genomes of the corresponding reference genomes used were downloaded from the NCBI website (https://www.ncbi.nlm.nih.gov; accessed on 30 August 2022). Genome quality was assessed through a re-implementation of an algorithm from an online tool (CheckM) [65] to ensure that genomes had acceptable completeness (≥95%) and contamination (≤5%), while any potential bacterial misassemblies were evaluated with the help of a web app (SkewIT) [66].

#### 2.4.2. Bioinformatics Analyses

All *L. monocytogenes* isolates were phenotypically characterized in silico from the obtained nucleotide sequences by using the microbial trait analyzer Traitar (GitHub Inc., San Francisco, CA, USA; https://github.com/aweimann/traitar, accessed on 6 February 2023) [67]. Additionally, all strains were molecularly confirmed through PCR-serogrouping and serotyping [68] performed with the Bionumerics platform version 8.1.1 (bioMérieux, Marcy l’ Etoile, France; https://www.applied-maths.com/bionumerics, accessed on 6 February 2023). The core/whole-genome MLST (cg/wg MLST) analyses, along with the assessment of acquired resistance, virulence factors, and screening for phage sequences, were also performed with Bionumerics version 8.1.1 (bioMérieux) for the in silico subtyping of the strains.

## 3. Results

### 3.1. Phenotypic Characterization of L. monocytogenes Isolates

**Figure 1:**
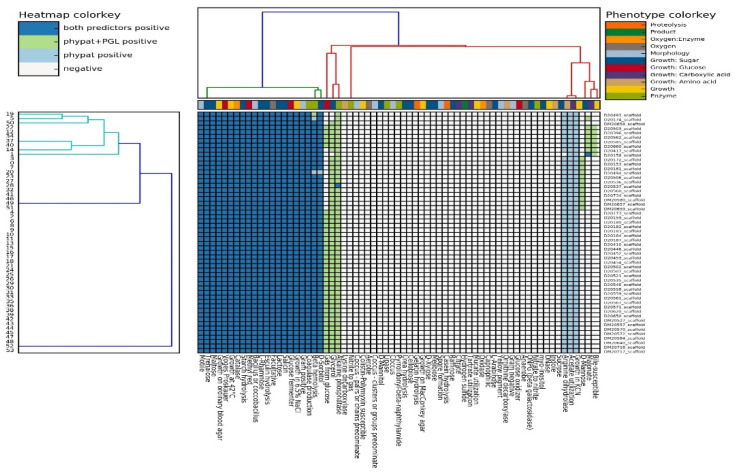
Phenotyping of *L. monocytogenes* with Traitar. Heatmap of phenotypic characteristics for the pathogen’s strains isolated from Myzithra soft whey cheese and related food processing surfaces. Key phenotypic characteristics for *L. monocytogenes* identification are grouped on the left side of the heatmap (dark blue entries). The color of the heatmap entries is determined by the origin of the phenotype’s prediction (Traitar phypat and/or phypat + PGL classifier). The colors of the dendrogram indicate similar phenotype distributions across strains, as determined by hierarchical clustering with SciPy (http://docs.scipy.org/doc/scipy/reference/cluster.hierarchy.html, accessed on 6 February 2023).

Fifty-four *L. monocytogenes* strains were isolated from Myzithra cheese (48 isolates) and related cheese processing surfaces (six isolates). Biochemical testing of the isolates (Supplementary Table S1) was consistent with the predicted phenotype for each *L. monocytogenes* strain, and all the phenotypic characteristics of the strains were mapped (Figure 1).

### 3.2. Antibiograms of L. monocytogenes Cheese and Surface Isolates

**Figure 2:**
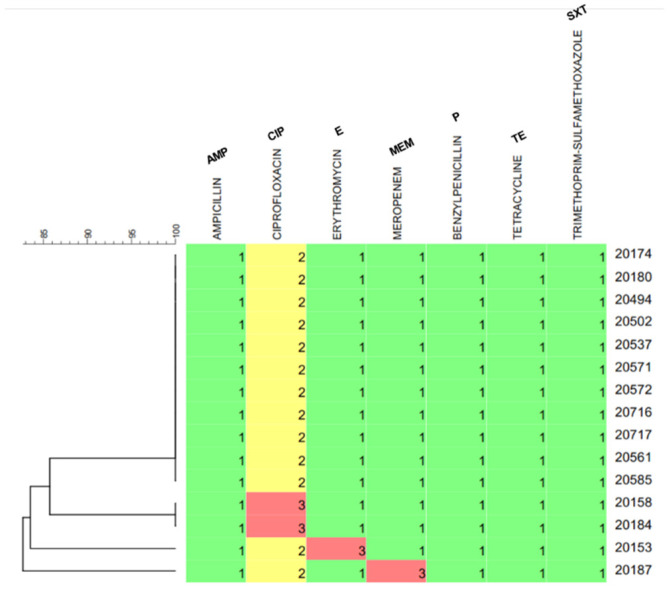
Antibiotic resistance profiles (antibiograms) of *L. monocytogenes* strains isolated from Myzithra soft whey cheese and related food processing surfaces. The number and color in the antibiogram for each strain designate its resistance to the selected antibiotic: (1) Green: Sensitive strain. (2) Yellow: Intermediate resistant strain. (3) Red: Resistant strain. The first eleven strains are a subsample from the microorganisms’ pool which displayed exactly the same antibiogram profile for better resolution of the dendrogram.

Seven antibiotics belonging to the classes of macrolides (E), tetracyclines (TE), penicillins (P, AMP), sulfonamides (SXT), fluoroquinolones (CIP), and carbapenems (MEM) were tested for the resistance of *L. monocytogenes* cheese and surface isolates against them. The antibiotic resistance profiles (antibiograms) of *L. monocytogenes* strains are presented in Figure 2. All isolates were not clearly susceptible to CIP and presented mostly intermediate (52/54 strains, 96.3% of isolates) to strong (2/54, 3.7%) resistance against the specific antimicrobial agent (Figure 2). CIP is not the drug of choice for the treatment of listeriosis and is not included in the clinical breakpoint tables for *L. monocytogenes* published by EUCAST [62]. Apart from CIP, therefore, all surface isolates were susceptible to the remaining six antibiotics tested, while 46 out of 48 (95.8%) of the cheese isolates were found to be sensitive during AST to those six antibiotics (Figure 2). None of the strains showed any multidrug resistance. In total, the resistance of four *L. monocytogenes* strains isolated from Myzithra soft cheese (4/54, 7.4%) was recorded against E, MEM and CIP (Figure 2). Three of the resistant isolates belonged to PCR-serogroup IIb (strains AAL 20153, AAL 20184, AAL 20187) and were classified as ST3 (CC3) strains, while the fourth resistant isolate belonged to PCR-serogroup IIa (strain AAL 20158) and was classified as an ST155 (CC155) strain (see Section 3.4). Furthermore, MIC values for the *L. monocytogenes* cheese isolates against E, CIP, CIP, and MEM were estimated at 0.19, 0.19, 0.50, and 0.64 μg/mL for strains AAL 20153, AAL 20184, AAL 20158, and AAL 20187, respectively. Following AST and MIC determination, strain AAL 20187 was finally designated as resistant to MEM.

Antibiotic resistance genes were identified in strain AAL 20158 and included *fosX*, *mdrL*, and *mdrM* genes conferring resistance to fosfomycin, QACs/macrolides and multidrug resistance, respectively. However, no resistance genes were identified to the rest of the isolates that exhibited antimicrobial resistance during AST.

### 3.3. Genetic Diversity of L. monocytogenes Isolates

**Figure 3:**
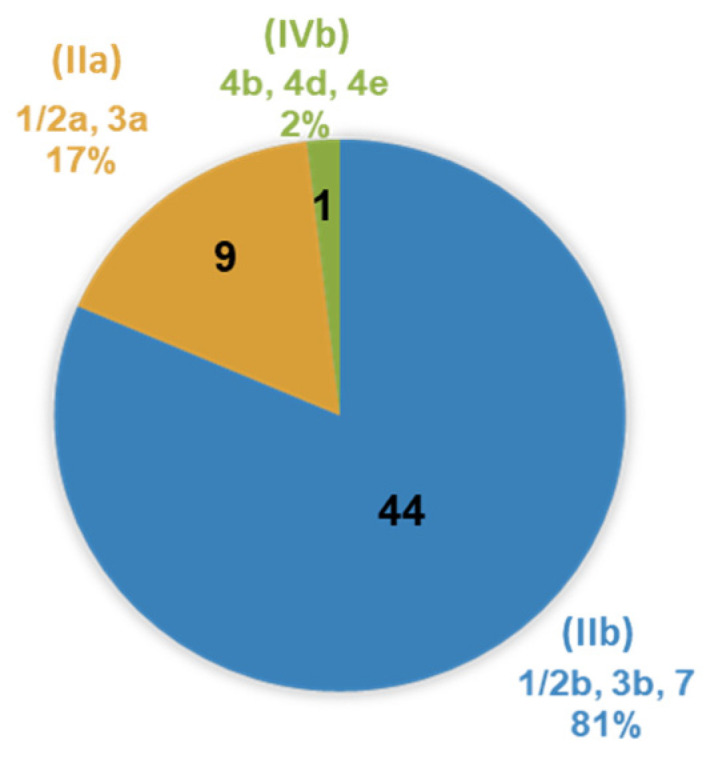
Distribution of PCR-serogroups and serotypes (ser.) of *L. monocytogenes* isolates recovered during microbiological monitoring of surfaces and end-products in a cheese processing facility. Distribution comprises of PCR-serogoups IIa: ser. 1/2a, 3a; IIb: ser. 1/2b, 3b, 7; IVb: ser. 4b, 4d, 4e, with the total number of strains and recovery percentages for each serogroup embedded in the chart.

PCR-serogrouping and serotyping performed for all the isolates revealed the presence of three major serogroups for the pathogen with their respective serotypes (i.e., PCR-serogroups IIa: ser. 1/2a, 3a; IIb: ser. 1/2b, 3b, 7; IVb: 4b, 4d, 4e) (Figure 3).

**Figure 4:**
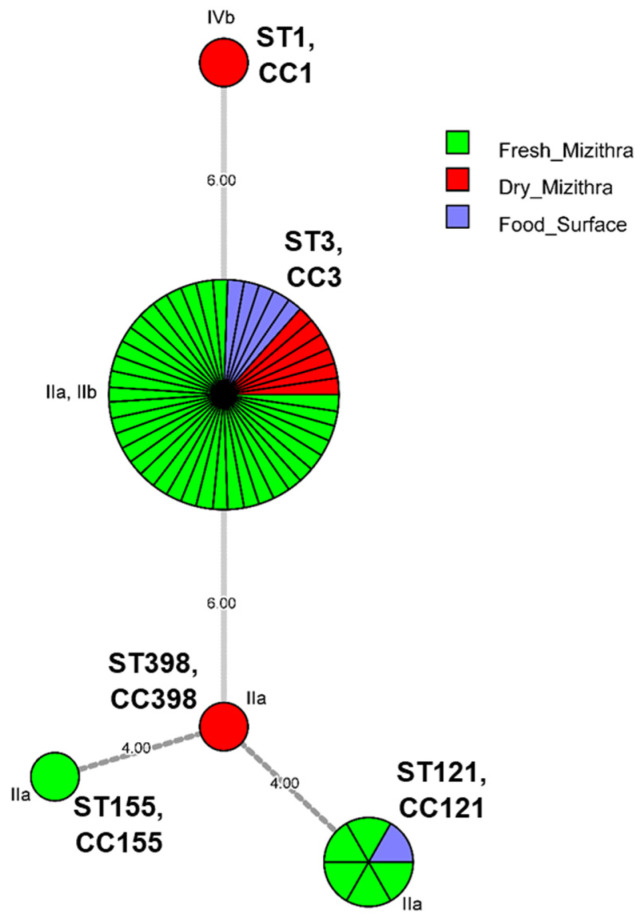
Minimum spanning tree (MST) based on MLST allelic profiles of *L. monocytogenes* isolates. The MST illustrates the phylogenetic relationship of the isolates, which are depicted in circles (each circle representing an ST and its homologous CC) with different colors (whether it is a surface or end-product isolate), the size of the circle being proportional to the number of strains belonging to the ST (CC). The circle fragments correspond to the different isolates. Links between the circles represent the number of allelic mismatches between STs (CCs).

**Figure 5:**
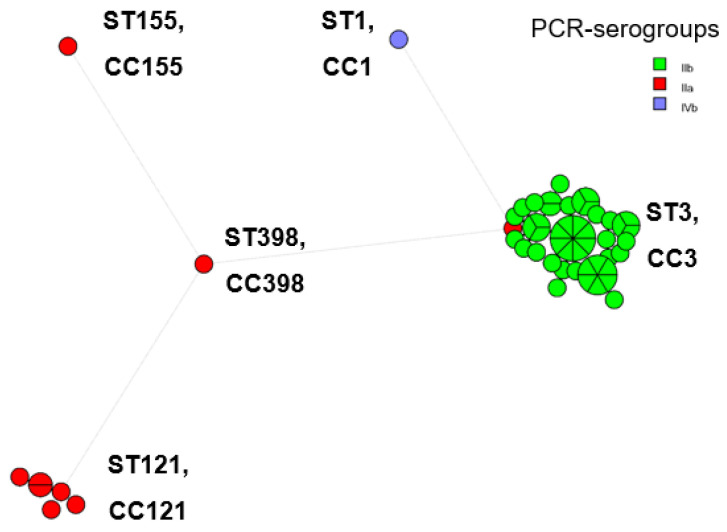
Minimum spanning tree (MST) based on cg/wgMLST (Core Pasteur Scheme) of *L. monocytogenes* isolates. This MST offers better resolution between similar strains compared to the MST presented in Figure 4.

A total of five different sequence types (STs) and clonal complexes (CCs) were identified in the 54 isolates of *L. monocytogenes* through the MLST (Figure 4) and cg/wg MLST analyses (Figure 5); ST3 and CC3 (each containing *n* = 45 strains, 83.3% of isolates), ST121 and CC121 (each *n* = 6, 11.0%), ST1 and CC1 (each *n* = 1, 1.9%), ST155 and CC155 (each *n* = 1, 1.9%), ST398 and CC398 (each *n* = 1, 1.9%). ST3 and CC3 strains belonged both to PCR-serogroups IIa and IIb with serotypes 1/2a, 3a and 1/2b, 3b, and 7 assigned to them, respectively. Strains of ST121 and CC121, ST155 and CC155, ST398 and CC398 all belonged to PCR-serogroup IIa and were assigned the serotypes 1/2a, 3a, whereas the ST1 and CC1 single strain belonged to PCR-serogroup IVb, which is comprised of serotypes 4b, 4d, 4e. The minimum spanning tree shows that the number of allelic differences between neighboring STs (CCs) ranged from 4 to 6 (Figure 4), but cg/wg MLST reveals genetic diversity between and within STs (CCs) (Figure 5).

### 3.4. Analysis of Resistance, Virulence and Persistence of L. monocytogenes Isolates

**Figure 6:**
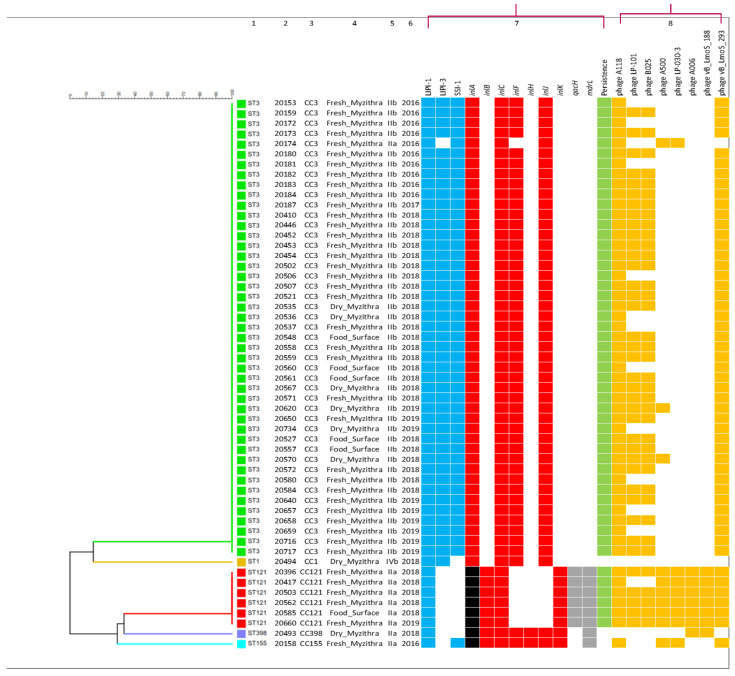
Dendrogram depicting the clustering of strains and clustering analysis of genes in *L. monocytogenes* isolates from Myzithra cheese and food processing surfaces. The heatmap shows the presence (color) or absence (no color) of the selected gene among the isolates. The black color indicates the presence of a truncated *inlA* gene. Column 1: Sequence type (ST), 2: Strain ID (AAL code), 3: Clonal complex (CC), 4: Isolation source (end-product or surface), 5: PCR-serogroup, 6: Year of strain isolation, 7: Detected genes and persistence trait, 8: Detected phages. Abbreviations for detected genes are given in the text.

The analysis performed with the Bionumerics software led us to focus on 12 important *Listeria* genes (Figure 6). Seven genes were related to acquired virulence potential (i.e., *inlA*, *inlB*, *inlC*, *inlF*, *inlH*, *inlJ*, and *inlK*), three corresponded to pathogenicity islands found in the bacterium (i.e., LIPI-1, LIPI-3, and SSI-1) and two genes were related with acquired resistance mechanisms (i.e., *qacH* and *mdrL*) against quaternary ammonium compounds (QACs) commonly used as disinfectants in the food industry.

All strains of PCR-serogroup IIa (with the notable exception of strain AAL 20174, which did not possess the operon LIPI-3) had the internalin A (*inlA*) gene truncated (Figure 6), something that confers to the reduced virulence potential of those strains. Moreover, the PCR-serogroup IIa strains belonging to ST121 (CC121) possessed the *qacH* and *mdrL* (Figure 6) genes indicating acquired resistance to QACs.

ST3 (CC3) and ST121 (CC121) *L. monocytogenes* strains showed persistence in the food processing environment as they were continuously isolated (even for over 2.5 years) from end-product cheeses and cheese-making surfaces, while they also exhibited mobile phage elements inserted and incorporated into their DNA (prophages) (i.e., from 2 to 8 elements; Figure 6), which are considered to promote increased resistance and adaptation of the strains to the adverse environmental conditions encountered in the food production plant. In contrast, the PCR-serogroup IVb strain belonging to ST1 (CC1) did not exhibit any such prophages at all and was more or less sporadic, indicative of a rather transient than persistent human contamination (Figure 6).

## 4. Discussion

A total of 54 *L. monocytogenes* strains isolated from Myzithra soft cheese and swabs collected from food contact surfaces in a cheese processing plant were characterized phenotypically and genetically using a WGS approach. The phenotypic characterization of the isolates in silico was consistent with the biochemical testing performed on the presumptive colonies of the pathogen recovered from ALOA, regarding at least some basic traits utilized for the *L. monocytogenes* identification (Figure 1). This confirms the constant recovery of the pathogen from end-product and surface samples during routine microbiological monitoring for *L. monocytogenes* presence in the facility, which in turn indicates potential colonization of the cheese processing environment with different listerial STs [52].

The majority of isolates were classified as PCR-serogroup IIb strains (44/54, 81.5%; ser. 1/2b, 3b, 7), followed by PCR-serogroup IIa (9/54, 16.7%; ser. 1/2a, 3a) and PCR-serogroup IVb strains (1/54, 1.8%; ser. 4b, 4d, 4e). Hence, lineage I and II strains accounted for 83.3 and 16.7% of the isolates, respectively. Many studies have highlighted the dominance of PCR-serogroup IIa (lineage II) strains of *L. monocytogenes* over a variety of food matrices as compared to other serogroups [6,7,8,50,69,70,71,72,73]. Nevertheless, the aforementioned distribution of serogroups (with the predominance of molecular serogroup IIb; lineage I) is not uncommon and has been reported elsewhere [74,75,76,77]. Especially when it comes to meat and dairy products, PCR-serogroup IIb is most prevalent among the *L. monocytogenes* isolates [69,74,76].

**Figure 7:**
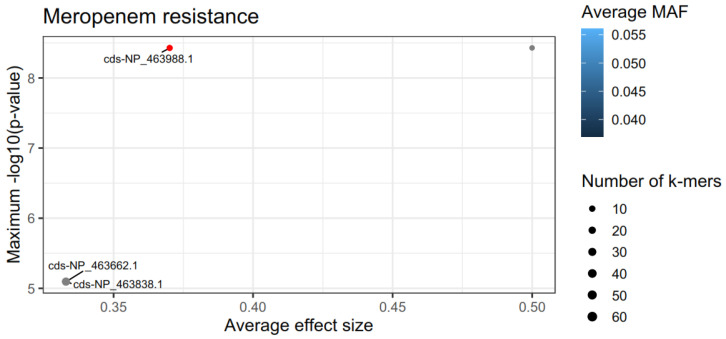
Summary plot obtained from GWAS analysis on the significant k-mers related to MEM-resistance of *L. monocytogenes* AAL 20187 strain.

Antibiotic resistance profiles for *L. monocytogenes* isolates from raw and/or pasteurized/fresh milk and milk products (e.g., cheeses) were revealed in previous research works against most of the antimicrobial agents tested in our study (i.e., TE, P, E, and SXT) [76,78,79,80]. Likewise, resistance to macrolides (E) emerged for strain AAL 20153 (Figure 2), although no resistance was recorded against tetracyclines, penicillins and sulfonamides in the present work. Furthermore, the MIC for E was found to range between 0.05 to 0.2 ppm in eight strains of *L. monocytogenes* tested for their antimicrobial resistance [81], something which agrees with our estimate of 0.19 ppm for the lethal concentration of the antibiotic in strain AAL 20153. The surface isolates belonged to two STs (ST3 and ST121; Figure 4) instead of the five STs identified for the cheese isolates (ST1, ST3, ST121, ST155, and ST398). However, no signs of antimicrobial resistance to any of the antibiotics used in the study were inferred for the surface isolates. All *L. monocytogenes* isolates showed no multidrug resistance, while the vast majority of them (50/54, 92.6%) were susceptible to all the antibiotics, except for CIP, to which generally all strains presented intermediate to strong resistance (Figure 2). The level of tolerance to the fluoroquinolone antibiotic CIP could be the result of cross-resistance developed in *L. monocytogenes*, when cells of the pathogen are exposed to sublethal concentrations of residual QACs, widely used as biocides in the food industry, leading to the formation of QAC-adapted subpopulations of *L. monocytogenes* with increased survival against CIP [82,83,84]. Interestingly, the resistance phenotype correlated with genotype in just one (strain AAL 20158) of the four *L. monocytogenes* strains initially screened as resistant to the single use of one of the three implicated antibiotics. On the other hand, no genes known to encode resistance against either E, CIP, or MEM were detected originally in the rest of the three resistant strains (strains AAL 20153, AAL 20184, and AAL 20187). Zhang et al. [85] described a similar situation where the authors could not detect by WGS any genes encoding resistance to MEM, against which antibiotic two clinical *L. monocytogenes* isolates showed resistance, suggesting that more research is needed on the matter. We hypothesize that this could be due to the possible fragmentation or mutation of the implicated resistance genes, and thus the necessity of using other search tools for gene alignment is highlighted in this case. For instance, *L. monocytogenes* genomes were studied with GWAS methodology (genome-wide association studies using pyseer) for the MEM-resistant phenotype, and the results are summarized in Figure 7. The top gene hit ranked by the −log10 (*p*-value), and the number of k-mer hits (cds-NP_463988.1; upper left) was related to a helix-turn-helix domain-containing protein with an average effect size of *ca.* 0.4 (0.370). MarR or Multiple antibiotics resistance-type regulators are characterized by winged helix-turn-helix (HTH) DNA-binding motifs and control genes that confer resistance to antibiotics, organic solvents, detergents, etc. This implies that this gene might have a significant role in assisting *L. monocytogenes* AAL 20187 resistance to MEM. However, other *L. monocytogenes* isolates may also possess this gene. The k-mer methodology showed regions in the sequence that are overrepresented in AAL 20187 compared to the rest of the strains.

The overwhelming majority of *L. monocytogenes* strains (51/54, 94.4%) belonged to CC3 and CC121 (Figure 4 and Figure 5), with those CCs commonly found among the food isolates [71,73,86,87]. All CC3 strains except one (strain AAL 20174) together with CC1 carried the hypervirulence pathogenicity island LIPI-3 in addition to LIPI-1 (Figure 6). On the contrary, LIPI-3 was absent in CC121, CC155 and CC398, strains of which CCs present a hypovirulent potential mainly because of the absence of the aforementioned major virulence factor LIPI-3 [88,89]. This hypovirulence is further expressed in CC121 strains of the pathogen due to the lack of internalins (i.e., *inlF*, *inlH*, *inlI*) noticed in the internalin gene family of the strains (Figure 6). Besides, the mutation in the *inlA* gene that leads to premature stop codons (PMSCs) has been found for CC121, CC155, and CC398 (Figure 6). It is now known that hypervirulent clones of *L. monocytogenes*, particularly CC1, are strongly associated with dairy products [87], whereas isolates of the pathogen carrying a virulence-attenuated mutation in *inlA* gene are frequently recovered from food processing environments [90]. Alarmingly, the isolation of the hypervirulent CC1 and CC3 strains from fresh and dry Myzithra as well as cheese processing surfaces, could represent a major public health issue, especially due to the ST1 strain’s close relation to dairy cattle farms [91]. Indeed, it is generally accepted that hypervirulent clones of *L. monocytogenes* colonize better the intestinal tract and more easily infect humans than the hypovirulent ones [87]. However, it is worthy of comment that hypo- or hypervirulence does not necessarily imply that the strain is capable of adhering to the human colon or effectively invading the host since foodborne diseases, such as listeriosis, are the result of the interaction between the host, the pathogen and the food commodity where the microorganism resides [92]. In that essence, many times, even hypovirulent clones of *L. monocytogenes* are able to cause infection in YOPI individuals.

Stress survival islet 1 (SSI-1) is comprised of a five-gene islet which contributes to the growth of *L. monocytogenes* under unfavorable conditions and allows for the pathogen’s survival in food processing environments [93]. SSI-1 was present only in the hypervirulent CC3 strains and the hypovirulent strain AAL 20158 (CC155), which is indicative of the good adaptive response these strains showed to the environment of the cheese processing plant, reflecting the constant recovery and, in absolute numbers, the higher persistence of CC3 strains in the facility (Figure 6). The persistence of *L. monocytogenes* in the facility was also evident for the hypovirulent strains of CC121 containing the QAC-resistant *qacH* and *mdrL* genes and harboring highly conserved plasmids and prophages [94]. CC121 is widely suspected of benzalkonium chloride (BC) tolerance [70,87,95], and strains of this cluster are normally equipped with the transposon Tn*6188*, which encodes, among others, the *qacH* gene conferring potential BC resistance [96]. In CC121, the mobile phage elements were at least six and reached up to eight elements for most of the strains as compared to any other CC (Figure 6). The *Listeria* phages detected in our study were among those used in a comparative genomic analysis of prophages conducted by Vu et al. [97].

Bioinformatics analysis revealed the presence of the competence transcription factor *comK* for all CC3 strains. *L. monocytogenes* strains carrying the SSI-1 cluster of genes as well as the *comK* prophage are more likely to possess a comparatively higher biofilm-forming ability and present enhanced persistence than strains of the pathogen lacking those genes [95,98], something that still has to be elucidated for our strain collection in terms of cell density and biofilm production for the CC3 strains. In addition, future research is to be focused on the single-nucleotide polymorphism (SNP) differences between the isolates in order to determine the genetic evolution of *L. monocytogenes* over the years in the cheese processing facility through the observed SNP differences caused by the limited single-nucleotide mutations but rapid diversification of prophages [99]. Facility-specific molecular markers utilized from the allelic variations detected across the whole genome of the pathogen’s isolates may be unique for specific processing plants and could be used as signatures for tracking *L. monocytogenes* throughout the food chain [100]. This has yet to be clarified in this instance. Incorporating cgMLST fingerprinting data obtained by WGS under the umbrella of a single tool used for the comparison of the whole genome of *L. monocytogenes* across distinct geographical regions is quite challenging. Recently a web-based platform has been proposed for tracking the pathogen worldwide to help with public health surveillance and resolution of foodborne disease outbreak investigations [101].

Notably, CC155 is mostly identified among *L. monocytogenes* isolates of clinical origin [85,87]. However, a WGS analysis showed that the specific CC was most prevalent among the pathogen’s isolates recovered from food and food production environments in Poland [102]. As far as CC398 is concerned, the only strain contained in this group (strain AAL 20493) warrants further investigation due to the limited information provided in the literature regarding this CC.

The present study provides insights into the pathogenic potential and persistence of the *L. monocytogenes* isolates recovered from a cheese processing facility. The findings of this work should be useful as they could be utilized for epidemiological investigations of *L. monocytogenes* in the food processing environment, revealing possible contamination scenarios, acquired antimicrobial resistance along the food production chain and providing critical information for post-outbreak management in the facility [103,104,105]. These findings also suggest that further research is needed for definitive genome mapping of the isolates.

## Data Availability

This Whole Genome Shotgun project (BioProject number PRJNA858978) has been deposited at DDBJ/ENA/GenBank under the accession numbers JANLLB000000000 to JANLLZ000000000, JANLMA000000000 to JANLMZ000000000, and JANLNA000000000 to JANLNC000000000. The version described in this paper is version JANLLB010000000 to JANLLZ010000000, JANLMA010000000 to JANLMZ010000000, and JANLNA010000000 to JANLNC010000000.

## Supplementary Materials

The following supporting information can be downloaded at: https://www.mdpi.com/article/10.3390/foods12061200/s1, Table S1: *Listeria monocytogenes* strains used in the study.
