# Supplementary material for: Characterization and Antibiotic Resistance of Listeria monocytogenes Strains Isolated from Greek Myzithra Soft Whey Cheese and Related Food Processing Surfaces over Two-and-a-Half Years of Safety Monitoring in a Cheese Processing Facility
Source: Foods. 2023 Mar 12;12(6):1200. doi: 10.3390/foods12061200 (PMC10048787; doi:10.3390/foods12061200)
Supplement: Supplementary file 1 [file foods-12-01200-s001.zip › foods-2237507-supplementary.pdf]

foods MDPI

Article

Characterization and Antibiotic Resistance of *Listeria monocytogenes* Strains Isolated from Greek Myzithra Soft Whey Cheese and Related Food Processing Surfaces Over Two- and Half-Years of Safety Monitoring in a Cheese Processing Facility

Nikolaos D. Andritsos [1] and Marios Mataragas [2,*]

[1] Department of Food Science and Technology, School of Agricultural Sciences, University of Patras, 2 G. Seferi Str., GR-301 00 Agrinio, Greece
[2] Department of Dairy Research, Institute of Technology of Agricultural Products, Hellenic Agricultural Organization "DEMETER", 3 Ethnikis Antistaseos Str., GR-452 21 Ioannina, Greece
* Correspondence: mmatster@gmail.com; Tel.: +30 26510 94780-9

Supplementary Material

Table S1. *Listeria monocytogenes* strains used in the study.

Strain No.	AAL code[1]	PCR-sero-group[2]	Serotype[2]	Sample	Sampling date	Oxidase test	Catalase reaction	Motility at 25 °C	Hemolysis on sheep blood agar	CAMP test	L-Rhamnose utilization	D-Xylose utilization
1	20153	IIb	1/2b, 3b, 7	Fresh Myzithra cheese	11.11.2016	-[3]	+[4]	+	+	SA+/RE-[5]	+	-
2	20158	IIa	1/2a, 3a	Fresh Myzithra cheese	11.11.2016	-	+	+	+	SA+/RE-	+	-
3	20159	IIb	1/2b, 3b, 7	Fresh Myzithra cheese	11.11.2016	-	+	+	+	SA+/RE-	+	-
4	20172	IIb	1/2b, 3b, 7	Fresh Myzithra cheese	09.12.2016	-	+	+	+	SA+/RE-	+	-
5	20173	IIb	1/2b, 3b, 7	Fresh Myzithra cheese	09.12.2016	-	+	+	+	SA+/RE-	+	-
6	20174	IIa	1/2a, 3a	Fresh Myzithra cheese	09.12.2016	-	+	+	+	SA+/RE-	+	-
7	20180	IIb	1/2b, 3b, 7	Fresh Myzithra cheese	20.12.2016	-	+	+	+	SA+/RE-	+	-

8	20181	IIb	1/2b, 3b, 7	Fresh Myzithra cheese	20.12.2016	-	+	+	+	SA+/RE-	+	-
9	20182	IIb	1/2b, 3b, 7	Fresh Myzithra cheese	20.12.2016	-	+	+	+	SA+/RE-	+	-
10	20183	IIb	1/2b, 3b, 7	Fresh Myzithra cheese	20.12.2016	-	+	+	+	SA+/RE-	+	-
11	20184	IIb	1/2b, 3b, 7	Fresh Myzithra cheese	20.12.2016	-	+	+	+	SA+/RE-	+	-
12	20187	IIb	1/2b, 3b, 7	Fresh Myzithra cheese	10.01.2017	-	+	+	+	SA+/RE-	+	-
13	20396	IIa	1/2a, 3a	Fresh Myzithra cheese	27.01.2018	-	+	+	+	SA+/RE-	+	-
14	20410	IIb	1/2b, 3b, 7	Fresh Myzithra cheese	16.03.2018	-	+	+	+	SA+/RE-	+	-
15	20417	IIa	1/2a, 3a	Fresh Myzithra cheese	27.03.2018	-	+	+	+	SA+/RE-	+	-
16	20446	IIb	1/2b, 3b, 7	Fresh Myzithra cheese	11.05.2018	-	+	+	+	SA+/RE-	+	-
17	20452	IIb	1/2b, 3b, 7	Fresh Myzithra cheese	29.05.2018	-	+	+	+	SA+/RE-	+	-

18	20453	IIb	1/2b, 3b, 7	Fresh Myzithra cheese	29.05.2018	-	+	+	+	SA+/RE-	+	-
19	20454	IIb	1/2b, 3b, 7	Fresh Myzithra cheese	29.05.2018	-	+	+	+	SA+/RE-	+	-
20	20493	IIa	1/2a, 3a	Dried Myzithra cheese	03.08.2018	-	+	+	+	SA+/RE-	+	-
21	20494	IVb	4b, 4d, 4e	Dried Myzithra cheese	03.082018	-	+	+	+	SA+/RE-	+	-
22	20502	IIb	1/2b, 3b, 7	Fresh Myzithra cheese	10.08.2018	-	+	+	+	SA+/RE-	+	-
23	20503	IIa	1/2a, 3a	Fresh Myzithra cheese	10.08.2018	-	+	+	+	SA+/RE-	+	-
24	20506	IIb	1/2b, 3b, 7	Fresh Myzithra cheese	10.08.2018	-	+	+	+	SA+/RE-	+	-
25	20507	IIb	1/2b, 3b, 7	Fresh Myzithra cheese	21.08.2018	-	+	+	+	SA+/RE-	+	-
26	20521	IIb	1/2b, 3b, 7	Fresh Myzithra cheese	21.08.2018	-	+	+	+	SA+/RE-	+	-
27	20527	IIb	1/2b, 3b, 7	Swab from surface No. 8	08.10.2018	-	+	+	+	SA+/RE-	+	-

28	20535	IIB	1/2b, 3b, 7	Dried Myzithra cheese	12.10.2018	-	+	+	+	SA+/RE-	+	-
29	20536	IIb	1/2b, 3b, 7	Dried Myzithra cheese	19.10.2018	-	+	+	+	SA+/RE-	+	-
30	20537	IIb	1/2b, 3b, 7	Fresh Myzithra cheese	19.10.2018	-	+	+	+	SA+/RE-	+	-
31	20548	IIb	1/2b, 3b, 7	Swab from surface No. 26	19.10.2018	-	+	+	+	SA+/RE-	+	-
32	20557	IIb	1/2b, 3b, 7	Swab from surface No. 3	26.10.2018	-	+	+	+	SA+/RE-	+	-
33	20558	IIb	1/2b, 3b, 7	Fresh Myzithra cheese	15.11.2018	-	+	+	+	SA+/RE-	+	-
34	20559	IIb	1/2b, 3b, 7	Fresh Myzithra cheese	23.11.2018	-	+	+	+	SA+/RE-	+	-
35	20560	IIb	1/2b, 3b, 7	Swab from surface No. 6AM	23.11.2018	-	+	+	+	SA+/RE-	+	-
36	20561	IIb	1/2b, 3b, 7	Swab from surface No. 1M	23.11.2018	-	+	+	+	SA+/RE-	+	-
37	20562	IIa	1/2a, 3a	Fresh Myzithra cheese	23.11.2018	-	+	+	+	SA+/RE-	+	-

38	20567	IIb	1/2b, 3b, 7	Dried Myzithra cheese	27.11.2018	-	+	+	+	SA+/RE-	+	-
39	20570	IIb	1/2b, 3b, 7	Dried Myzithra cheese	4.12.2018	-	+	+	+	SA+/RE-	+	-
40	20571	IIb	1/2b, 3b, 7	Fresh Myzithra cheese	11.12.2018	-	+	+	+	SA+/RE-	+	-
41	20572	IIb	1/2b, 3b, 7	Fresh Myzithra cheese	11.12.2018	-	+	+	+	SA+/RE-	+	-
42	20580	IIb	1/2b, 3b, 7	Fresh Myzithra cheese	11.12.2018	-	+	+	+	SA+/RE-	+	-
43	20584	IIb	1/2b, 3b, 7	Fresh Myzithra cheese	11.12.2018	-	+	+	+	SA+/RE-	+	-
44	20585	IIa	1/2a, 3a	Swab from surface No. 4	18.12.2018	-	+	+	+	SA+/RE-	+	-
45	20620	IIb	1/2b, 3b, 7	Dried Myzithra cheese	18.12.2018	-	+	+	+	SA+/RE-	+	-
46	20640	IIb	1/2b, 3b, 7	Fresh Myzithra cheese	01.03.2019	-	+	+	+	SA+/RE-	+	-
47	20650	IIb	1/2b, 3b, 7	Fresh Myzithra cheese	09.04.2019	-	+	+	+	SA+/RE-	+	-

48	20657	IIb	1/2b, 3b, 7	Fresh Myzithra cheese	03.05.2019	-	+	+	+	SA+/RE-	+	-
49	20658	IIb	1/2b, 3b, 7	Fresh Myzithra cheese	15.05.2019	-	+	+	+	SA+/RE-	+	-
50	20659	IIb	1/2b, 3b, 7	Fresh Myzithra cheese	15.05.2019	-	+	+	+	SA+/RE-	+	-
51	20660	IIa	1/2a, 3a	Fresh Myzithra cheese	15.05.2019	-	+	+	+	SA+/RE-	+	-
52	20716	IIb	1/2b, 3b, 7	Fresh Myzithra cheese	15.05.2019	-	+	+	+	SA+/RE-	+	-
53	20717	IIb	1/2b, 3b, 7	Fresh Myzithra cheese	02.07.2019	-	+	+	+	SA+/RE-	+	-
54	20734	IIb	1/2b, 3b, 7	Dried Myzithra cheese	02.07.2019	-	+	+	+	SA+/RE-	+	-

[1] AAL: Eurofins Athens Analysis Laboratories S.A.; Microbiology Laboratory, Metamorfosi, Attica, Greece.

[2] PCR-serogroup and serotype designation of *L. monocytogenes* performed *in silico* using Bionumerics version 8.1.1. (https://www.applied-maths.com/bionumerics).

[3] -: negative reaction/result.

[4] +: positive reaction/result.

[5] SA+/RE-: positive reaction with *Staphylococcus aureus* and negative reaction with *Rhodococcus equi* (CAMP test).

Disclaimer/Publisher's Note: The statements, opinions and data contained in all publications are solely those of the individual author(s) and contributor(s) and not of MDPI and/or the editor(s). MDPI and/or the editor(s) disclaim responsibility for any injury to people or property resulting from any ideas, methods, instructions or products referred to in the content.
